# Supplementary material for: Candida expansion in the gut of lung cancer patients associates with an ecological signature that supports growth under dysbiotic conditions
Source: Nat Commun. 2023 May 9;14:2673. doi: 10.1038/s41467-023-38058-8 (PMC10169812; doi:10.1038/s41467-023-38058-8)
Supplement: Supplementary file 1 — Supplementary Information [file 41467_2023_38058_MOESM1_ESM.pdf]

Supplementary Information

Supplementary Note 1

Detailed information on SCFA producers

We found that many of the bacterial species predictive of **LC** with high robustness were short-chain fatty acid (SCFA) producers [1–6], including *Bifidobacterium adolescentis, Eubacterium rectale* [6], *Anaerotruncus colihominis* [2,6], *Alistipes ihumii AP11* [5], several *Lachnospiraceae* species, *Pseudoflavonifractor capillosus*, and *Odoribacter splanchnicus*. Less robust predictors involved in SCFA production include *Roseburia inulinivorans* and *Alistipes finegoldii*.

SCFA producers in the human colon are typically from the phylum Firmicutes [6]. The most abundant SCFAs in human stool are butyrate, propionate, and acetate [7]. SCFAs are commonly produced by anaerobic fermentation of otherwise non-digestible fiber [4,7]. Hence, most SCFA producing microbes are strict or obligate anaerobes. However, while fermentation routes from carbohydrates are understood well, other routes (e.g., from amino acids) are less understood.

There is a distinction between *primary degraders* (*e.g.*, species being able to ferment specific polysaccharides to mono, di- and oligosaccharides to acetate and lactate) and *secondary degraders* (i.e., species that use degraded and fermented output from primary degraders and metabolize them into more new compounds such as butyrate) [4,8].

B. adolescentis is a obligate anaerobe and considered a primary degrader (resistant starch) [4,8]. Bifidobacteria process the uncommon fructose-6-phosphate phosphoketolase pathway, which is used to ferment fructo-oligosaccharides [8]. It is a strong producer of acetate and is likely involved in cross-feeding butyrate producers [4,8].

Eubacterium species and *Roseburia* species are strict anaerobe, abundant, potent butyrate producer [4,6,7]. They are commonly associated with good health.

A. colihominis is a strict anaerobe and produces butyrate even in GAM medium [2].

Alistipes are obligate anaerobes and were shown to produce propionate and acetate [5]. In general, *Alistipes* species, including *A. finegoldii*, are often found decreased in disease individuals with inflammatory bowel disease (IBD) and non-alcoholic fatty-liver (NAFLD) [5]. *Alistipes ihumii* AP 11 has only recently been sequenced and is less researched.

P. capillosus contains a homologue of the butyrate kinase enzyme [2] and isolates from chicken gut were able to produce butyrate [1].

O. splanchnicus is a strict anaerobe and potent butyrate-producing species that associated with good health [3]. Furthermore, it presents anti-inflammatory properties [3]. While commonly identified, it is often less abundant than other butyrate producers (E. rectale, F. prausnitzii) [3].

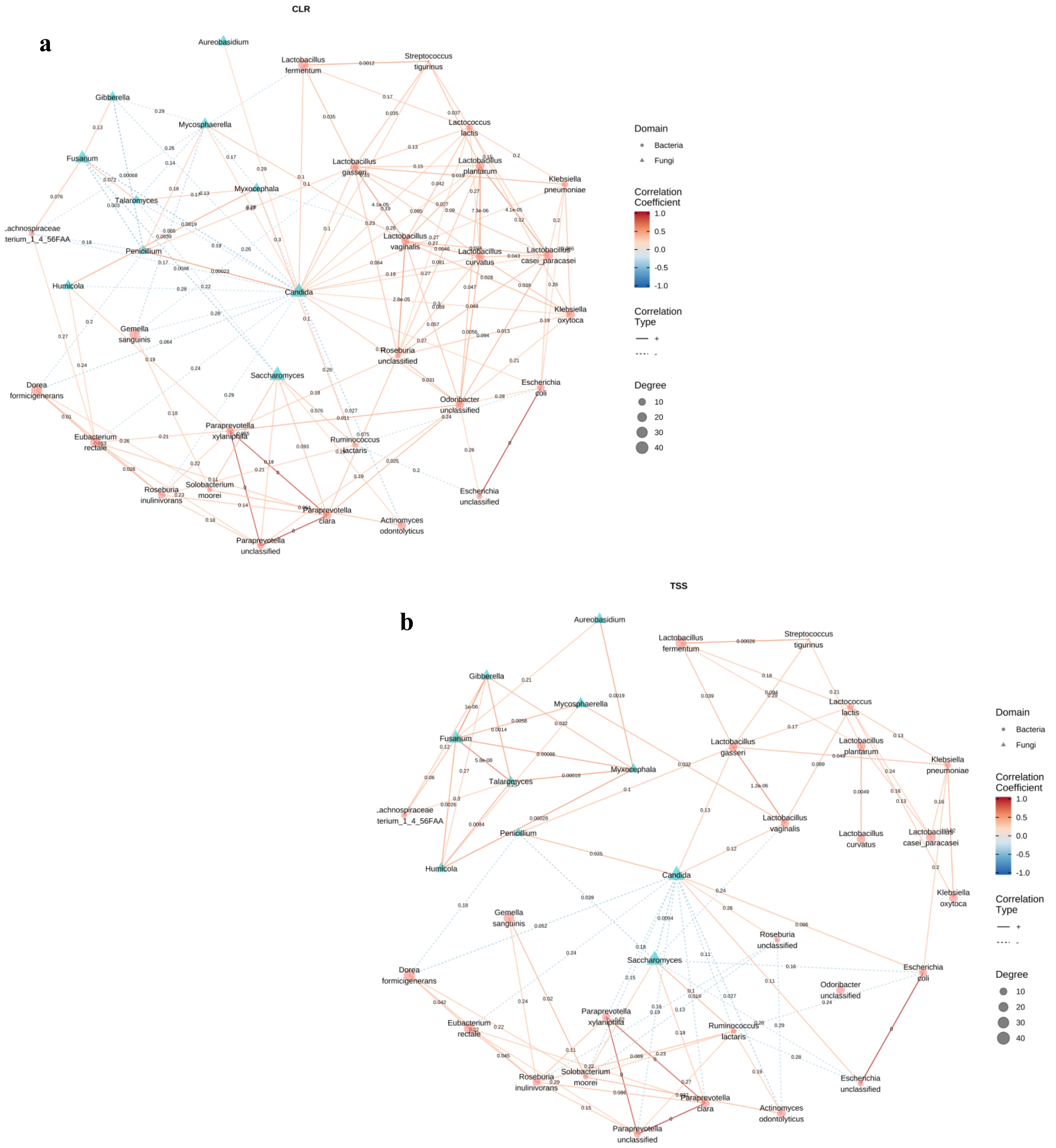

Figure S1. Trans-kingdom co-abundance networks of bacterial species and fungal genera abundances. Correlations were assessed using two-sided Spearman's coefficients on centre log ratio (CLR)-normalized (left) or total-sum scaled (TSS)-normalized abundance data (right). Only significant correlations with raw *P*<0.05 and |*r*|>10% are shown. Red, solid edges show positive correlations; blue-dashed edges show negative correlations. Colour strength indicates estimated correlation strength. Edge labels show the false-discovery-rate (FDR) correction of multiple testing. **(a)** CLR-based networks capture microbial abundance correlations better, especially when comparing features between distinct compositions. However, strong changes in observed relative abundance (TSS) were lost. **(b)** TSS networks revealed correlations between dominant species but were less reliable for low-abundance species. **(a-b)** *Lactobacillus* species such as *L. gasseri* and *L. lactis* and *Escherichia* species correlated positively with *Candida* regardless of normalization, and short-chain fatty acid producers like *Lachnospiraceae* and *Actinomyces odontolyticus* were found to correlate negatively.

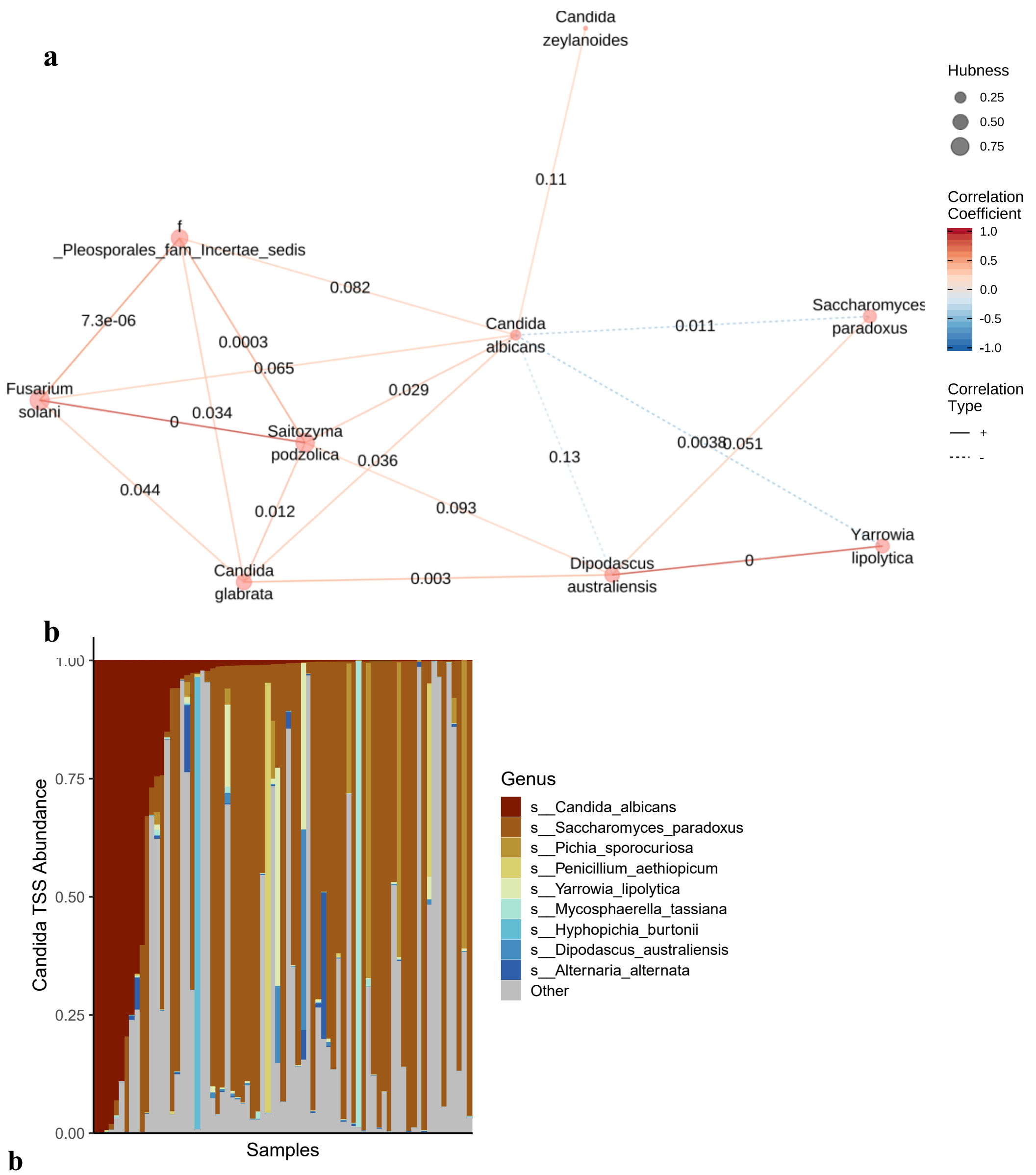

b

Figure S2. *Candida albicans* correlated negatively with *Yarrowia lipolytica* and *Saccharomyces paradoxus* abundance. **(a)** Fungal co-abundance networks of significant correlations (P<0.05; |r|>0.10; two-sided; Spearman's coefficient) with *C. albicans*. Solid, red lines indicate positive correlations. Dashed, blue lines indicate negative correlations. Edge labels show the false-discovery-rate (FDR) correction of multiple testing. **(b)** Fungal species abundance profile indicating the top 10 most abundant fungal species based on total-sum scaling. Species are shown in colours. Other fungi are grey.

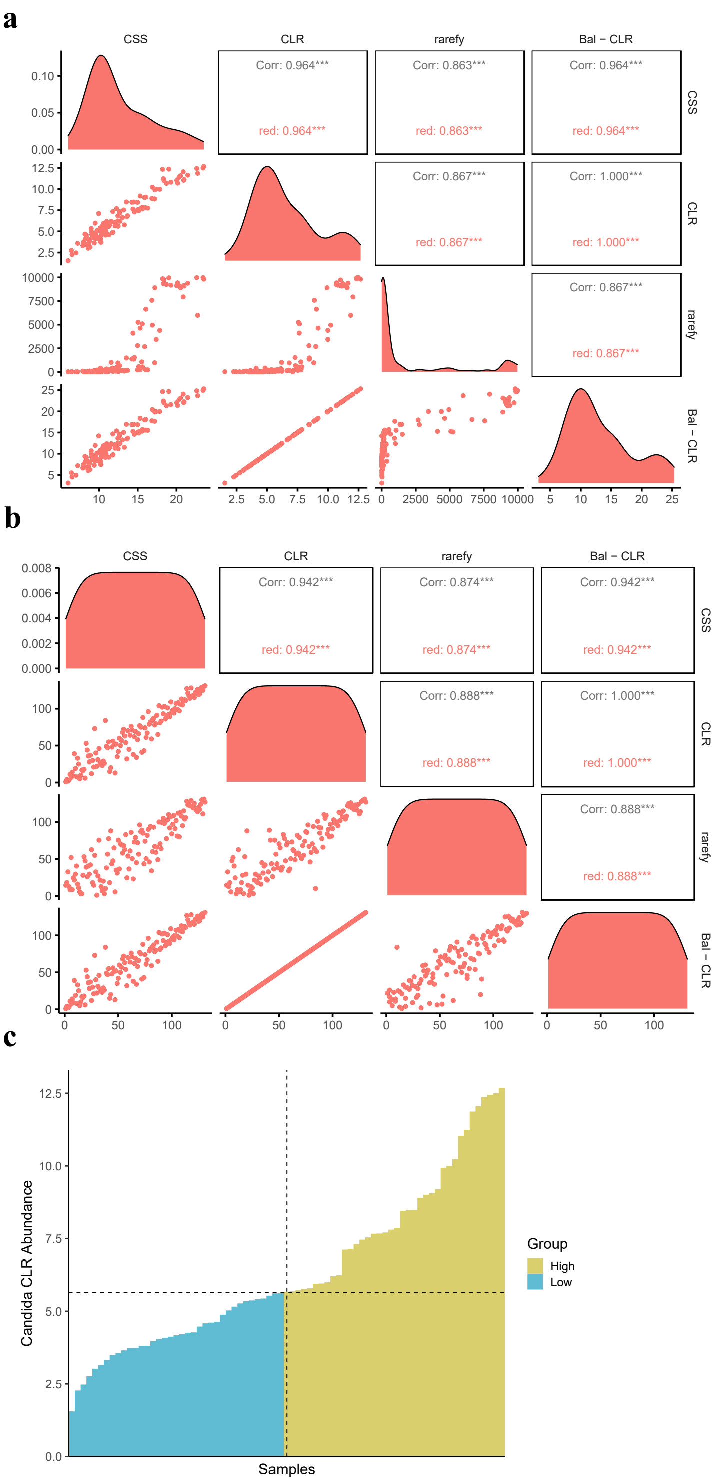

Figure S3. Normalised *Candida* abundance correlates across methods. In normalised space **(a)**, all methods except rarefaction showed acceptable linear correlation (cumulative sum scaling [CSS]; centralized log-ratio [CLR]; balance-based CLR [bal-CLR]). Rarefaction differs were resolved using a simple rank transformation **(b)**, implying at least a non-linear relationship among all methods. The strongest agreement was seen for CSS and CLR. Rarefaction was a poor normalisation choice, as it accounted only for differences in sequencing depth and not for compositionality effects in relative abundance data. **(c)** *Candida* CLR-normalised abundance (y-axis) per sample (x-axis). Samples are ordered from low to high CLR abundance. Dashed lines indicate median sample and median abundance thresholds. **(a-b)** Significance was assessed using two-sided Pearson correlation tests. Raw P-values are indicated with stars ($^{\star}P<0.05$, $^{\star\star}P<0.01$, $^{\star\star\star}P<0.001$).

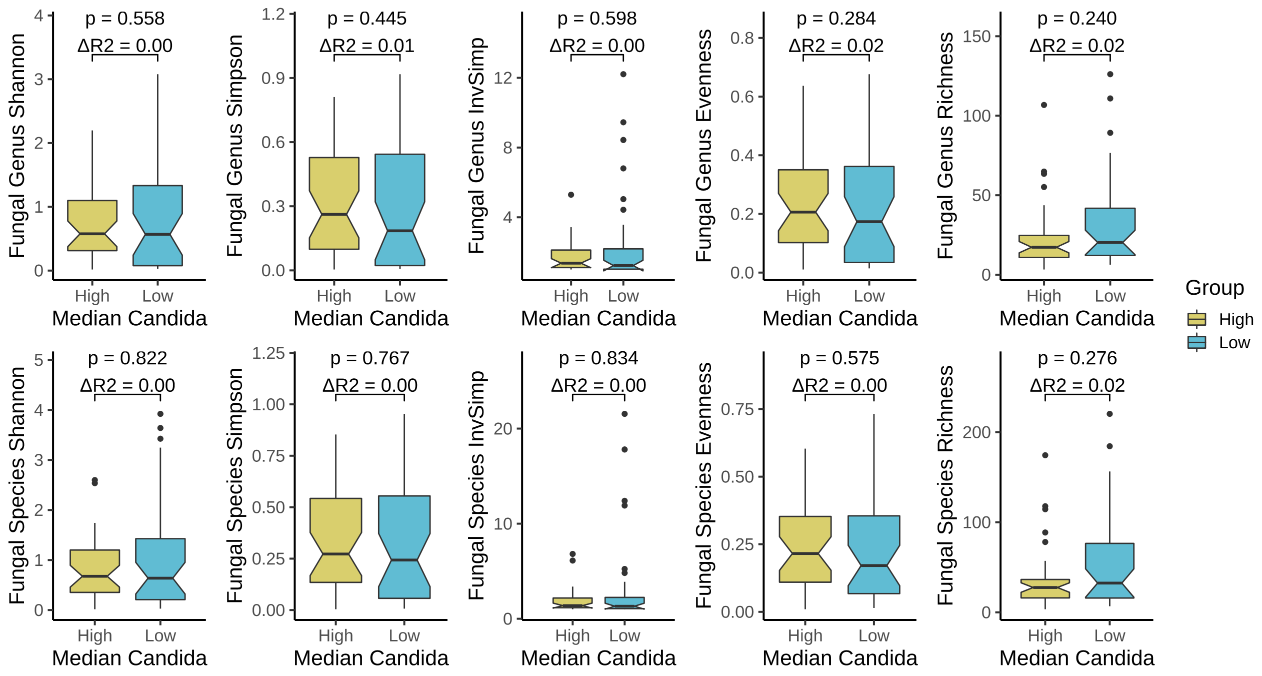

Figure S4. Fungal alpha diversity at genus (top-row) and species (bottom-row) level. 10 box plots over 5 different alpha diversity indices (by column) were computed. Centre lines denote the median value, boxes contain the Q1 and Q3 quartiles (IQR). The whiskers extend up to 1.5 × IQR, and values beyond these bounds are considered outliers. Significance test between high and low *Candida* samples was performed using two-sided Wilcoxon rank-sum test. None of the indices is significant (raw P>0.05).

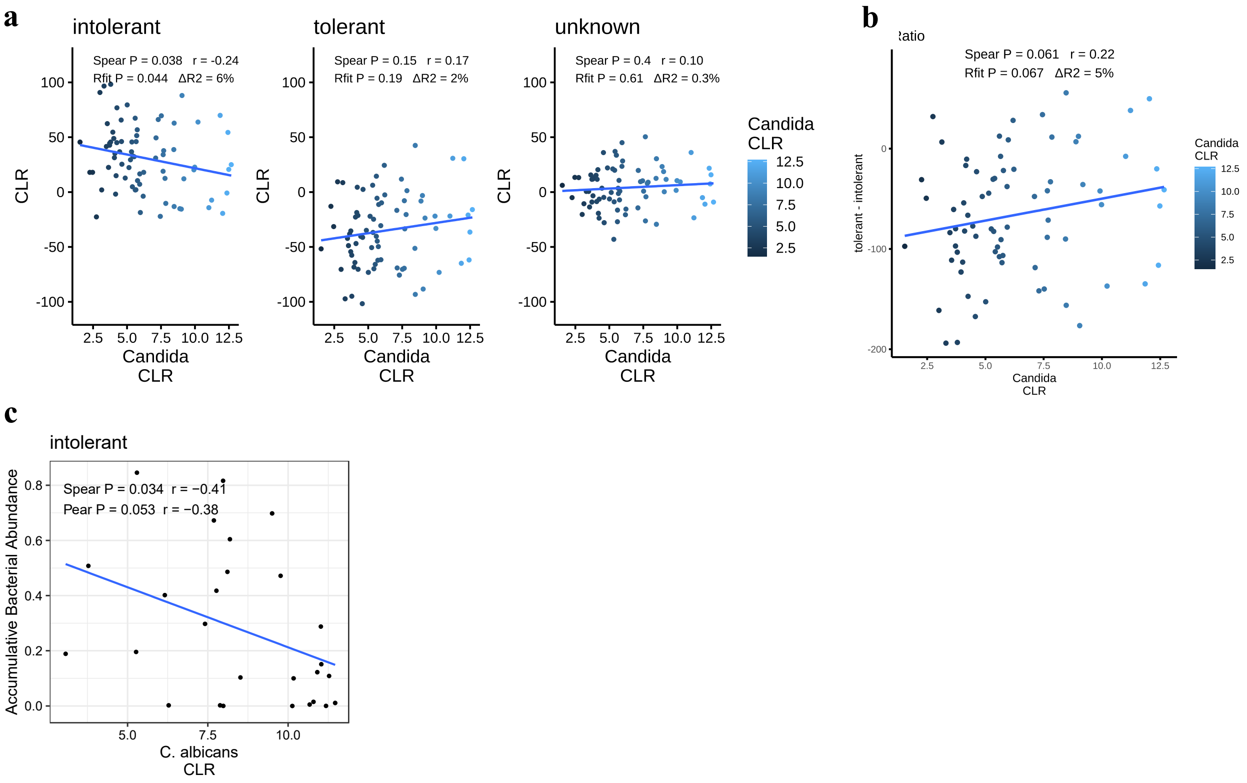

Figure S5. Correlation of *Candida* with abundance of bacteria stratified by oxygen-tolerance. Bacterial abundances were summed per sample based on the ability to grow in the presence of oxygen. **(a-b)** Results for main analysis cohort. Statistical tests were performed using (1) non-parametric GLM (Rfit) controlling for sex+age and (2) two-sided Spearman correlation test. Oxygen-intolerant bacteria (strict or obligate anaerobes) show decreased abundance for samples with high Candida abundance. **(c)** Additional cohort from patients in intensive care units (ICU) showing the same effect for *Candida albicans* [*unpublished data*]. Statistical comparisons were performed using two-sided Pearson and Spearman correlation tests. **(a-c)** Blue lines show the slope of a linear model (mean), and grey shadings indicates the 95% confidence interval.

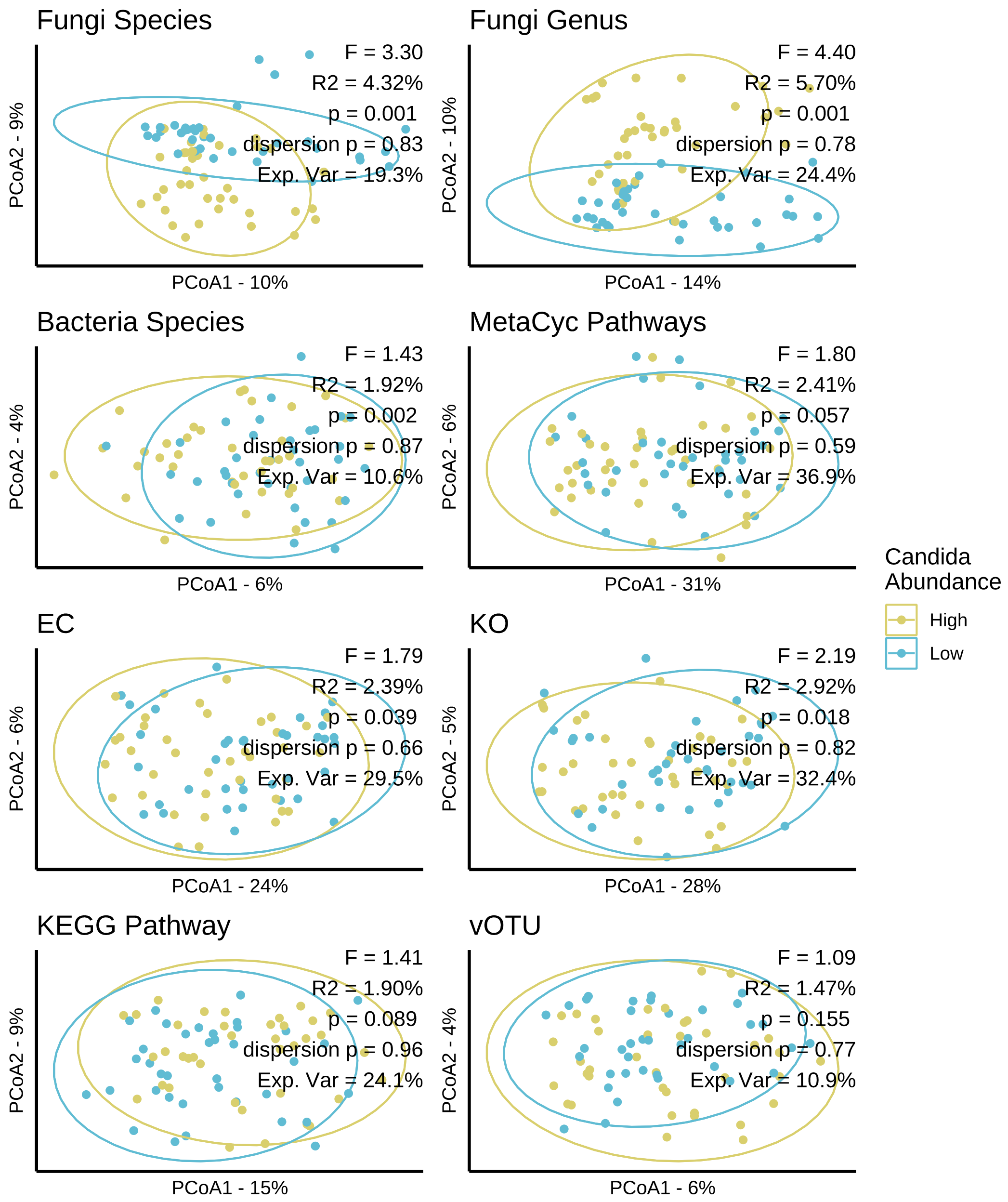

Figure S6. **Principal coordinate analysis (PCoA) of beta diversity for bacterial species and functional, fungal genus and species, and viral operational taxonomic unit abundances (vOTU)**. Statistical testing was performed using PERMANOVA. Raw *P* values are shown. R^2 describes estimated explained variance by *Candida* High vs. Low grouping. MetaCyc and KEGG Orthology had the largest effect sizes.

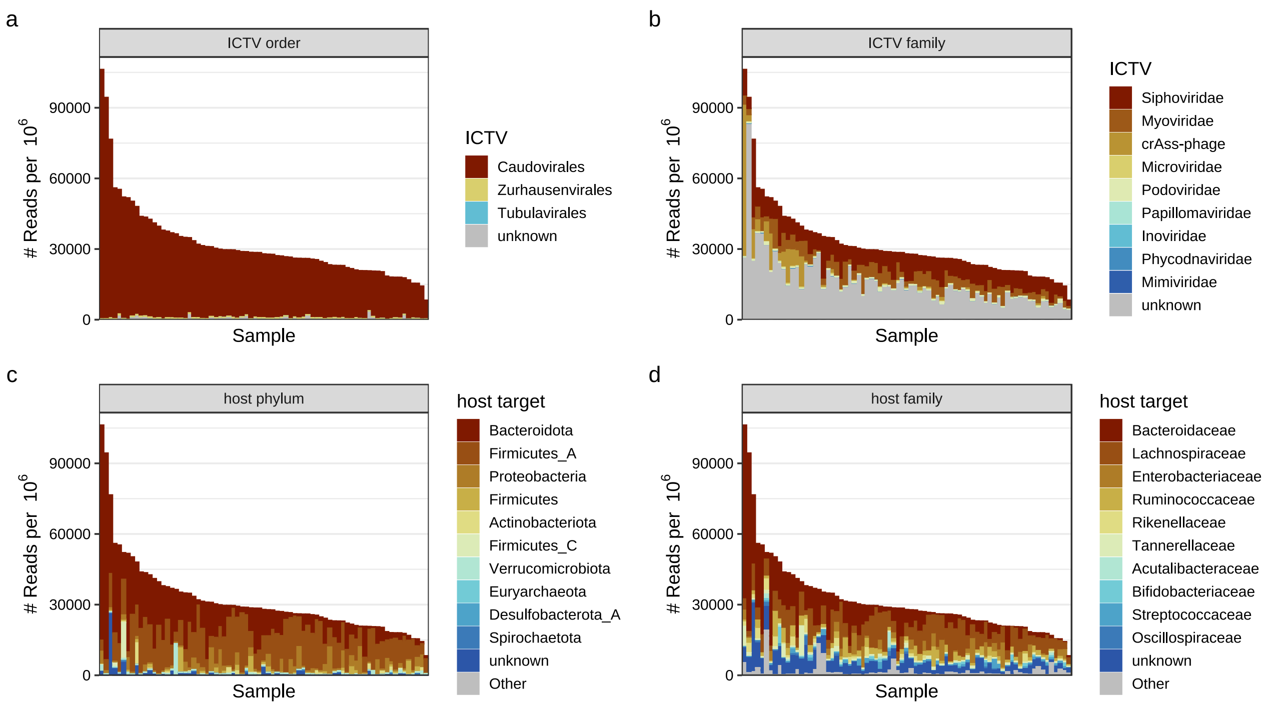

Figure S7. Virome composition using the MGV catalogue. Viral genome reads were summed-up according to (**a-b**) the International Committee on Taxonomy of Viruses (ICTV) and (**c-d**) predicted host-target bacterium. Most phages belong to the order Caudovirales or families Siphoviridae, Myoviridae and CrAss-phages. In line with the bacterial profile, the majority of phages target Bacteroidata and Firmicutes. Of note, host prediction is based on the GTDB annotation, which includes sub-phyla annotations (e.g., Firmicutes_A, Firmicutes_C).

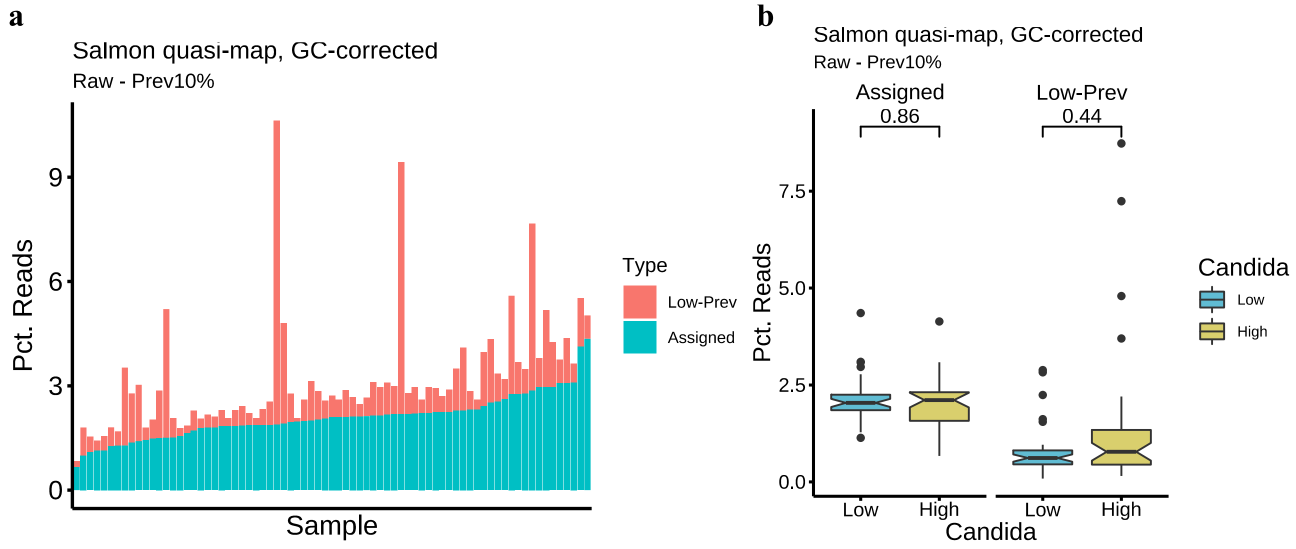

Figure S8. Metagenomic gut virus (MGV) phage mapping rate. (a) Percentage of reads per sample that passed (blue) or did not pass (red) a 10% prevalence filter. More than 30% of reads were assigned to low prevalence contigs in a few samples. **(b)** Boxplots showing the percentage of reads assigned to phage contigs by *Candida* High or Low abundance. Centre lines denote the median value, boxes contain the Q1 and Q3 quartiles (IQR). The whiskers extend up to 1.5 × IQR, and values beyond these bounds are considered outliers. Statistical significance was assessed by unpaired Wilcoxon rank-sum tests. The median 2.5% of reads were assigned to prevalent viral contigs.

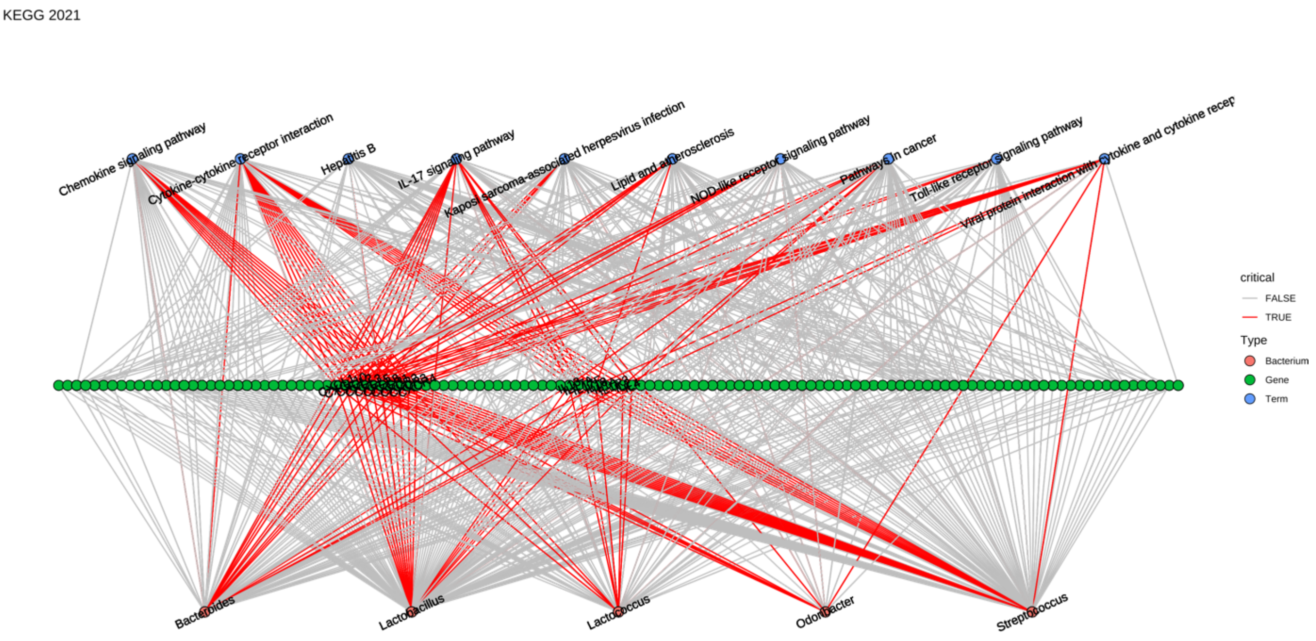

Figure S9. Species enriched in the High *Candida* (HC) group were related to human-disease genes. Microbial Set Enrichment Analysis revealed that bacterial genera with significant differential abundance in the HC group (bottom) were frequently associated with human disease genes (n=280) in contrast to the low *Candida* (LC) group (n=0). Green nodes indicate human disease-associated genes that were tested for significant enrichment in KEGG pathways (red nodes). Grey edges indicate an association between either (1) bacteria and host genes or (2) host genes and pathways. Red edges indicate associations with interleukins and chemokines. IL-17 signalling is of interest because of strong roles in immune cell recruitment after bacterial invasion and in tumour response.

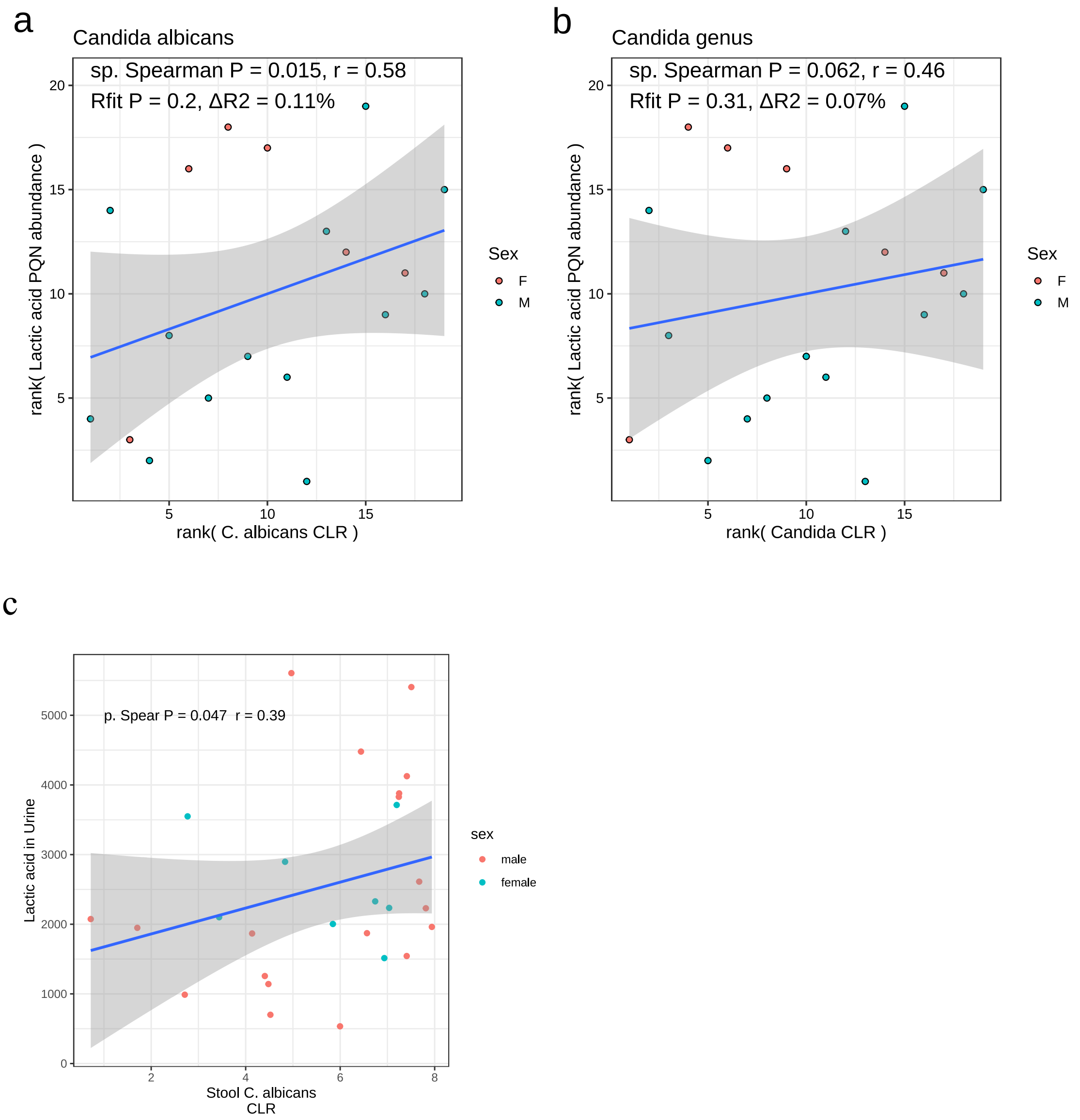

Figure S10. Metabolomics measurements of Lactate correlation with *Candida*. Species abundances were CLR normalized.
(a-b) Scatter plot comparing **urine** lactate abundance from patients of the main analysis cohort. Results for correlation with (a) *C. albicans* and (b) *Candida* genus CLR-normalized abundance. Abundances were rank-transformed prior to plotting. Significance assessed using partial Spearman controlling for differences in Sex+BMI.
(c) Scatter plot of blood **serum** lactate measurements from an independent cohort of ICU patients. Significance assessed using partial Spearman correlation controlling for differences in Sex [*unpublished data*].
(a-c) Blue lines show the slope of a linear model (mean), and grey shadings indicates the 95% confidence interval.

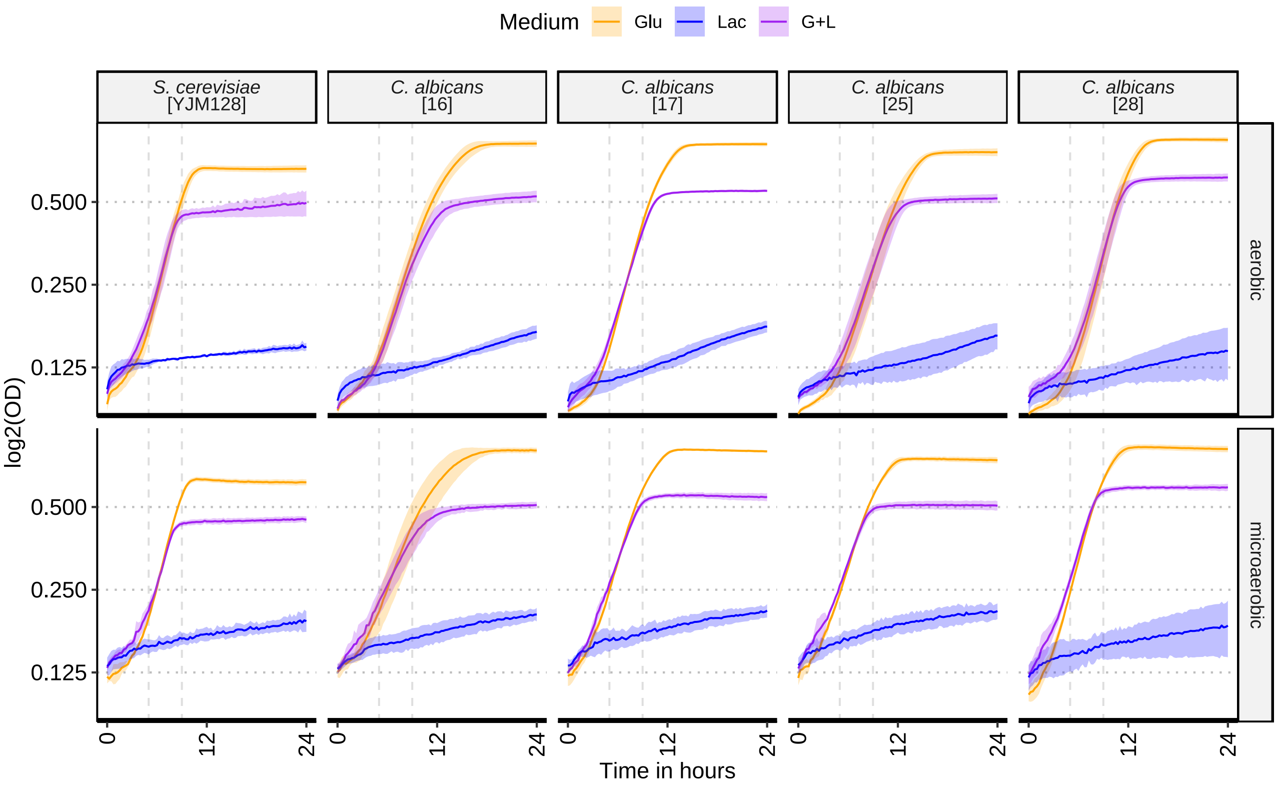

Figure S11. Monoculture grow rate. Growth rate of four *C. albicans* gut isolates (x-axis) and the human-associated pathogenic *Saccharomyces cerevisiae* strain YJM128 (left to right panels) under varying oxygen levels (aerobic - top panel; microaerobic - bottom panel) and carbon sources (only glucose – yellow; only lactate – blue; glucose+lactate – violet). Exponential growth phase for most strains (except under sole lactate) lied between 5h and 11.5h of culturing. Lines depict the mean, ribbons the standard deviation, using four biological replicates.

References

1. Eeckhaut, V. *et al.* Butyrate production in phylogenetically diverse Firmicutes isolated from the chicken caecum. *Microb. Biotechnol.* **4**, 503 (2011).

2. Gotoh, A. *et al.* Use of Gifu Anaerobic Medium for culturing 32 dominant species of human gut microbes and its evaluation based on short-chain fatty acids fermentation profiles. *Biosci. Biotechnol. Biochem.* **81**, 2009–2017 (2017).

3. Hiippala, K. *et al.* Novel Odoribacter splanchnicus Strain and Its Outer Membrane Vesicles Exert Immunoregulatory Effects in vitro. *Front. Microbiol.* **11**, (2020).

4. Baxter, N. T. *et al.* Dynamics of human gut microbiota and short-chain fatty acids in response to dietary interventions with three fermentable fibers. *MBio* **10**, (2019).

5. Parker, B. J., Wearsch, P. A., Veloo, A. C. M. & Rodriguez-Palacios, A. The Genus Alistipes: Gut Bacteria With Emerging Implications to Inflammation, Cancer, and Mental Health. *Front. Immunol.* **11**, 906 (2020).

6. Louis, P. & Flint, H. J. Diversity, metabolism and microbial ecology of butyrate-producing bacteria from the human large intestine. *FEMS Microbiol. Lett.* **294**, 1–8 (2009).

7. Louis, P. & Flint, H. J. Formation of propionate and butyrate by the human colonic microbiota. *Environ. Microbiol.* **19**, 29–41 (2017).

8. Belenguer, A. *et al.* Two Routes of Metabolic Cross-Feeding between Bifidobacterium adolescentis and Butyrate-Producing Anaerobes from the Human Gut. *Appl. Environ. Microbiol.* **72**, 3593 (2006).
