# Supplementary material for: Candida expansion in the gut of lung cancer patients associates with an ecological signature that supports growth under dysbiotic conditions
Source: Nat Commun. 2023 May 9;14:2673. doi: 10.1038/s41467-023-38058-8 (PMC10169812; doi:10.1038/s41467-023-38058-8)
Supplement: Supplementary file 3 — Description of Additional Supplementary Files [file 41467_2023_38058_MOESM3_ESM.docx]

# Description of Additional Supplementary Files

**File Name: Supplementary Data 1
Description:** Statistical test results for significant differences in alpha and beta diversity between groups.

**File Name: Supplementary Data 2
Description:** Differential abundance test results of bacteria, fungal, MetaCyc pathways, Pathway enrichment (GSAE), contribution diversity (MetaCyc Contri), Enzyme commission (EC) and viral operational taxonomic unit (vOTU) abundances.

**File Name: Supplementary Data 3
Description:** Genome-scale metabolic modelling diets, and results for short-chain fatty acids from different diets and oxygen levels.

**File Name: Supplementary Data 4
Description:** Microbial set enrichment results (MSEA).

**File Name: Supplementary Data 5
Description:** Growth rates and competition experiment measurements.

**File Name: Supplementary Data 6
Description:** Patient characteristics, grouping cut-offs, and *Candida* albicans isolates.
