# Supplementary material for: Candida expansion in the gut of lung cancer patients associates with an ecological signature that supports growth under dysbiotic conditions
Source: Nat Commun. 2023 May 9;14:2673. doi: 10.1038/s41467-023-38058-8 (PMC10169812; doi:10.1038/s41467-023-38058-8)
Supplement: Supplementary file 10 — Reporting Summary [file 41467_2023_38058_MOESM10_ESM.pdf]

nature portfolio

Corresponding author(s): Gianni Panagiotou

Last updated by author(s): Mar 16, 2023

Reporting Summary

Nature Portfolio wishes to improve the reproducibility of the work that we publish. This form provides structure for consistency and transparency in reporting. For further information on Nature Portfolio policies, see our Editorial Policies and the Editorial Policy Checklist.

Statistics

For all statistical analyses, confirm that the following items are present in the figure legend, table legend, main text, or Methods section.

n/a	Confirmed	
☐	☒	The exact sample size (*n*) for each experimental group/condition, given as a discrete number and unit of measurement
☐	☒	A statement on whether measurements were taken from distinct samples or whether the same sample was measured repeatedly
☐	☒	The statistical test(s) used AND whether they are one- or two-sided<br>*Only common tests should be described solely by name; describe more complex techniques in the Methods section.*
☐	☒	A description of all covariates tested
☐	☒	A description of any assumptions or corrections, such as tests of normality and adjustment for multiple comparisons
☐	☒	A full description of the statistical parameters including central tendency (e.g. means) or other basic estimates (e.g. regression coefficient) AND variation (e.g. standard deviation) or associated estimates of uncertainty (e.g. confidence intervals)
☐	☒	For null hypothesis testing, the test statistic (e.g. *F*, *t*, *r*) with confidence intervals, effect sizes, degrees of freedom and *P* value noted<br>*Give P values as exact values whenever suitable.*
☒	☐	For Bayesian analysis, information on the choice of priors and Markov chain Monte Carlo settings
☒	☐	For hierarchical and complex designs, identification of the appropriate level for tests and full reporting of outcomes
☐	☒	Estimates of effect sizes (e.g. Cohen's *d*, Pearson's *r*), indicating how they were calculated

Our web collection on statistics for biologists contains articles on many of the points above.

Software and code

Policy information about availability of computer code

Data collection	Sequencing data produced using Illumina sequencing platform (HiSeq2500 2x250bp).
Data analysis	Open source software: Sunbeam 2.1.0, Humann2 0.11.1, Salmon 1.5.2<br><br>Ecology and statistical analysis environment:<br>R 4.1<br>R packages: tidyverse 1.3.0, DADA2 1.20, vegan 2.5-7, ggplot2 3.3.3, GGally 2.1.2, igraph 1.2.6, ggraph 2.0.5, tidygraph 1.2.0, ggsignif 0.6.1, Rfit 0.24.2, maaslin2 1.4.0, zCompositions 1.3.4, SIAMCAT 1.12.0, SpiecEasi 1.1.0<br><br>GSMM Analysis Environment: python 3.6.8, IBM CPLEX 12.8.0.0<br>Python packages: COBRApy 0.17.1, Pandas 0.23.4<br><br>MAMBO Environment: python 3.7, IBM CPLEX 12.8.0.0<br><br>Machine Learning Code is available at a GitHub repository (doi.org/10.5281/zenodo.7730477)

For manuscripts utilizing custom algorithms or software that are central to the research but not yet described in published literature, software must be made available to editors and reviewers. We strongly encourage code deposition in a community repository (e.g. GitHub). See the Nature Portfolio guidelines for submitting code & software for further information.

Data

Policy information about availability of data

All manuscripts must include a data availability statement. This statement should provide the following information, where applicable:

- Accession codes, unique identifiers, or web links for publicly available datasets
- A description of any restrictions on data availability
- For clinical datasets or third party data, please ensure that the statement adheres to our policy

Sequencing data generated and analyzed during the current study are available in the NCBI SRA repository as Bio Project PRJNA811494 (ncbi.nlm.nih.gov/bioproject/PRJNA81)

HHUMAnN2 databases are available under:
ChoCoPhlAn (huttenhower.sph.harvard.edu/humann2_data/chocophlan/full_chocophlan.v201901.tar.gz),
UniRef90 (huttenhower.sph.harvard.edu/humann2_data/uniprot/uniref_annotated/uniref90_annotated_v201901.tar.gz)
KEGG and E.C. mapping (huttenhower.sph.harvard.edu/humann2_data/full_mapping_1_1.tar.gz)
The Metagenomic Gut Virus (MGV) v1.0 2021_07_08 (https://portal.nersc.gov/MGV/MGV_v1.0_2021_07_08.tar.gz)
Fungal UNITE database release 01-12-2017 (https://doi.org/10.15156/BIO/587475)
Metabolomis data is available from MetaboLights (XXX)

AGORA models (https://www.vmh.life/files/reconstructions/AGORA/1.03/AGORA-1.03-With-Mucins.zip)
CarveMe collections (https://github.com/cdanielmachado/embl_gems/tree/master/models)
Standard diets:
https://vmh.life/#nutrition
western and high fiber diets:
https://static-content.springer.com/esm/art%3A10.1038%2Fnbt.3703/MediaObjects/41587_2017_BFnbt3703_MOESM2_ESM.pdf (Supplementary Table 12)

Human research participants

Policy information about studies involving human research participants and Sex and Gender in Research.

Reporting on sex and gender	We acquired information on sex (biological) based on self-reports of patients and personal identification cards. We did not acquire information regarding gender. We did not identify systematic differences in sex for top-level investigations. For investigating sex-specific changes with high-dimensional features, our study with n=75 patients is likely underpowered. Patient-wise information on sex are submitted alongside our study.<br><br>Cohort #1 had n=75 patients, with n=44 males and n=31 females (41%).<br>Cohort #2 had n=27 patients, with n=19 males and n=8 females (30%).
Population characteristics	The main study cohort (#1) entails patients who underwent diagnostic pulmonary procedures in 2018, including Stage IIIB/IV NSCLC (n=68) and other lung cancer histologies (n=7). Lung cancer patients were treated with anti-PD-1 immune checkpoint inhibitors, including nivolumab or pembrolizumab. Clinicopathological parameters including sex (m=44, f=31), age (68±5), body mass index (26.3±4.2), alcohol usage(never=35, current=9, former=6, occasionally=25), histology (Adenocacinoma=40, Squamous=28, Other=7), chronic obstructive pulmonary disease (with=41, without=34), line of treatment (1st=16, 2nd=42, 3rd=15, 4th=2) and response to therapy (R=27, NR=48) were recorded. The clinical stage was assessed based on the Union for International Cancer Control (8th edition), and age was recorded at the time of diagnosis. Response to therapy was evaluated according to RECIST 1.1.<br><br>The secondary validation cohort (#2) entails non-cancer, intensive care unit (ICU) patients (cohort #2; n=27) from an on-going study. A total of n=22 with signs of systemic infection were treated with broad-spectrum antibiotics (piperacillin/tazobactam=18, meropenem=3). Clinicopathological parameters including sex (m=19, f=8), age (67±11), body mass index (28.6±5.1), SOFA (9.8±3), sepsis (with=19, without=8), diet (oral=5, tube-fed=22), and mechanical ventilation (with=23, without=4) were recorded at time of hospitalization and during stay at ICU.
Recruitment	According to our study's inclusion criteria (main study cohort #1), we are not aware of any decision to participate in our study that is left entirely up to individuals. Consecutive non-preselected patient treated with advanced-stage NSCLC were invited to participate in 2018 and gave their signed and informed consent. Patient were recruited at the National Koranyi Institute of Pulmonology, Budapest, Hungary and at the County Hospital of Pulmonology, Torokbalint, Hungary. With a high participation rate, we are not aware of any self-selection bias that systematically influenced our inclusion criteria.<br><br>For the secondary validation cohort (#2), intensive care unit (ICU) patients without cancer treated at the University Hospital Jena, Germany, were invited to participate and give signed and informed consent. Critically ill patients were included if either (a) without systematic antimicrobial therapy within the last 7 days and an expected ICU length of stay of more than 3 days, or (b) treated with meropenem or piperacillin/tazobactam started within the last 72 hours. We are not aware of any self-selection bias that systematically influenced our inclusion criteria.
Ethics oversight	This study on cohort #1 was approved by the national-level ethics committee (Hungarian Scientific and Research Ethics Committee of the Medical Research Council, (ETT-TUKEB-50302-2-2017-EKU)). The study of cohort #2 was approved by the

Ethics Committee of the Jena University Hospital, Germany (MS-ICU 2019-1306-Material). In both cohorts, all samples were collected from patients receiving standard of care treatments (SOC), the sample collection itself was not a therapeutic intervention and did not require listing on clinicaltrials.gov. Patients gave written, informed consent to participate in this study according to CARE guidelines and in compliance with the Declaration of Helsinki principles. After clinical information was collected, patient identifiers were removed so patients cannot be identified directly or indirectly.

Note that full information on the approval of the study protocol must also be provided in the manuscript.

Field-specific reporting

Please select the one below that is the best fit for your research. If you are not sure, read the appropriate sections before making your selection.

- [x] Life sciences
- [ ] Behavioural & social sciences
- [ ] Ecological, evolutionary & environmental sciences

For a reference copy of the document with all sections, see nature.com/documents/nr-reporting-summary-flat.pdf

Life sciences study design

All studies must disclose on these points even when the disclosure is negative.

Sample size	It is currently not possible to estimate the number of required patients for our proposed microbiota study. Published studies looking at fecal microbiota vary widely in sample size and do not indicate how the participant numbers were devised. Published studies on gut bacterial-fungal interactions in humans (based on feces samples) are rare, but varied from only 8 patients and 5 healthy controls (Zhai 2021, Nat. Med.) to 99 patients (Ricardo García-Gamboa 2021).<br><br>Based on previous studies, we feel that 75 samples (+11 for validation) represent a good middle-ground in sample size to investigate interactions of gut microbiota in vivo. No sample size calculation was performed, but our study cohort was big enough to identify significant differences in ecological properties.
Data exclusions	No data was excluded.
Replication	Each sample from the study cohort was sequenced using both ITS2 and whole metagenomic sequencing of the same DNA extraction producing overlapping microbiome profiles. Prediction of Candida (High, Low) levels using machine learning models were then validated in an independent validation cohort of cancer patients using the same sequencing procedures. Another cohort (#2) was used for replicating results with severely ill, but non-cancer, patients.<br><br>Further experiments (in vitro culturing) were performed 4 times (4 biological replicates) per experiment conditions. These replications were performed on different days. Measurement outcomes between experiments of the same condition were similar.
Randomization	As this was an observational study, there was no assignment of patients. Analyses were adjusted for covariates (gender, BMI) using a non-parametric generalized linear models (GLM; "Rfit") or regular GLMs
Blinding	Extractions were performed by a technician unfamiliar with the project design. After clinical information was collected, patient identifiers were removed so patients cannot be identified directly or indirectly. De-identified samples were send for metagenomic sequencing and used for downstream processing

Reporting for specific materials, systems and methods

We require information from authors about some types of materials, experimental systems and methods used in many studies. Here, indicate whether each material, system or method listed is relevant to your study. If you are not sure if a list item applies to your research, read the appropriate section before selecting a response.

Materials & experimental systems

n/a	Involved in the study	
[x]	[ ]	Antibodies
[x]	[ ]	Eukaryotic cell lines
[x]	[ ]	Palaeontology and archaeology
[x]	[ ]	Animals and other organisms
[x]	[ ]	Clinical data
[x]	[ ]	Dual use research of concern

Methods

n/a	Involved in the study	
[x]	[ ]	ChIP-seq
[x]	[ ]	Flow cytometry
[x]	[ ]	MRI-based neuroimaging
